# Supplementary material for: Integrative transcriptomic analysis reveals key drivers of acute peanut allergic reactions
Source: Nat Commun. 2017 Dec 5;8:1943. doi: 10.1038/s41467-017-02188-7 (PMC5715016; doi:10.1038/s41467-017-02188-7)
Supplement: Supplementary file 1 — Description of Additional Supplementary Files [file 41467_2017_2188_MOESM1_ESM.pdf]

Description of Additional Supplementary Files

File Name: Supplementary Data 1
Description: List of all peanut response genes.

File Name: Supplementary Data 2
Description: Gene ontology results for peanut response (blue) module.

File Name: Supplementary Data 3
Description: Gene ontology results for up and down regulated genes in the peanut response (blue) module.
