# Supplementary material for: Integrative transcriptomic analysis reveals key drivers of acute peanut allergic reactions
Source: Nat Commun. 2017 Dec 5;8:1943. doi: 10.1038/s41467-017-02188-7 (PMC5715016; doi:10.1038/s41467-017-02188-7)
Supplement: Supplementary file 2 — Supplementary Information [file 41467_2017_2188_MOESM2_ESM.pdf]

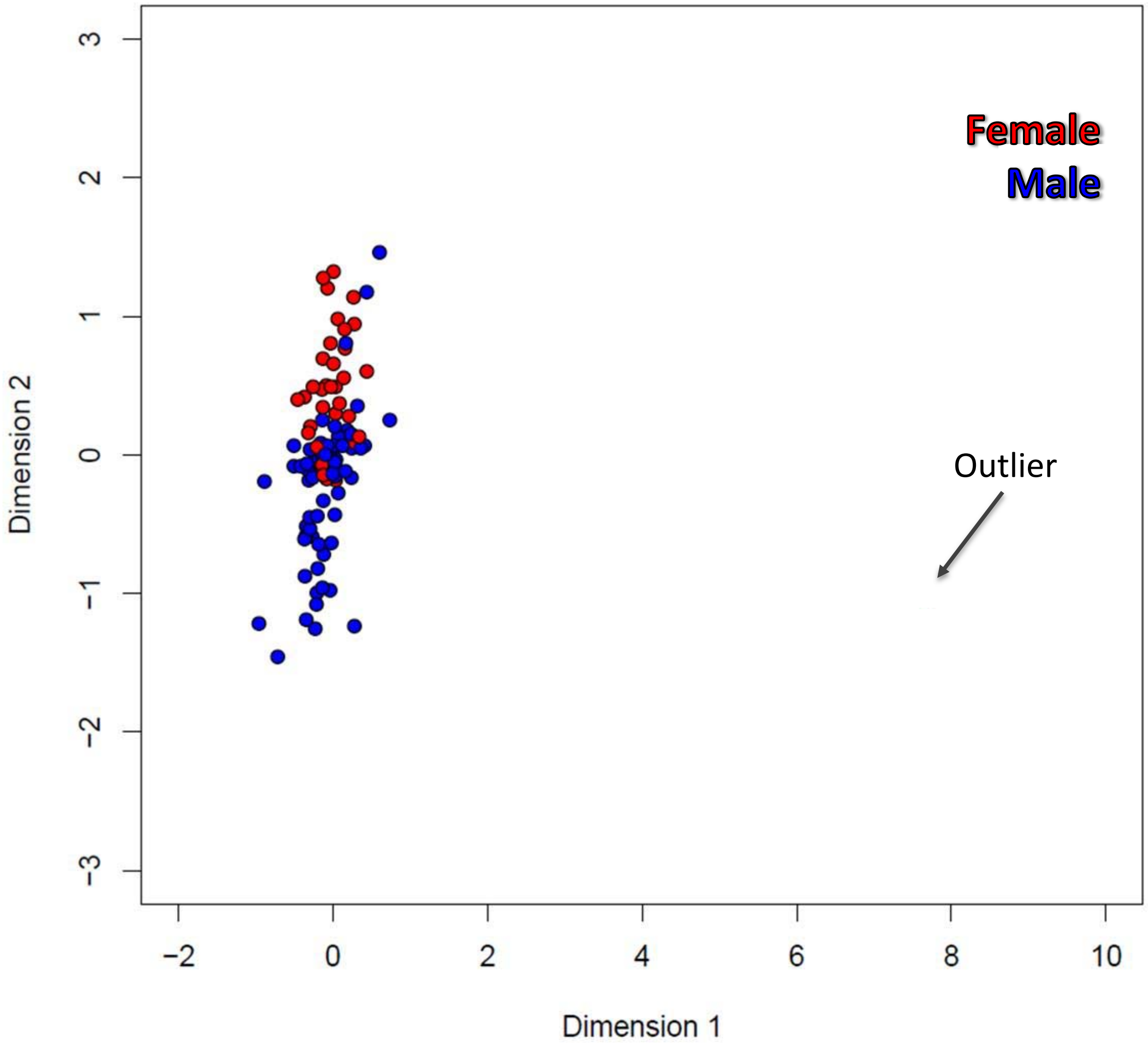

Supplementary Figure 1. **RNAseq QC outlier detection.** Plot depicting multidimensional scaling analysis to identify outlier RNA-seq samples in the discovery cohort (n=19). Points are labeled according to gender, and the single outlier sample is indicated (arrow).

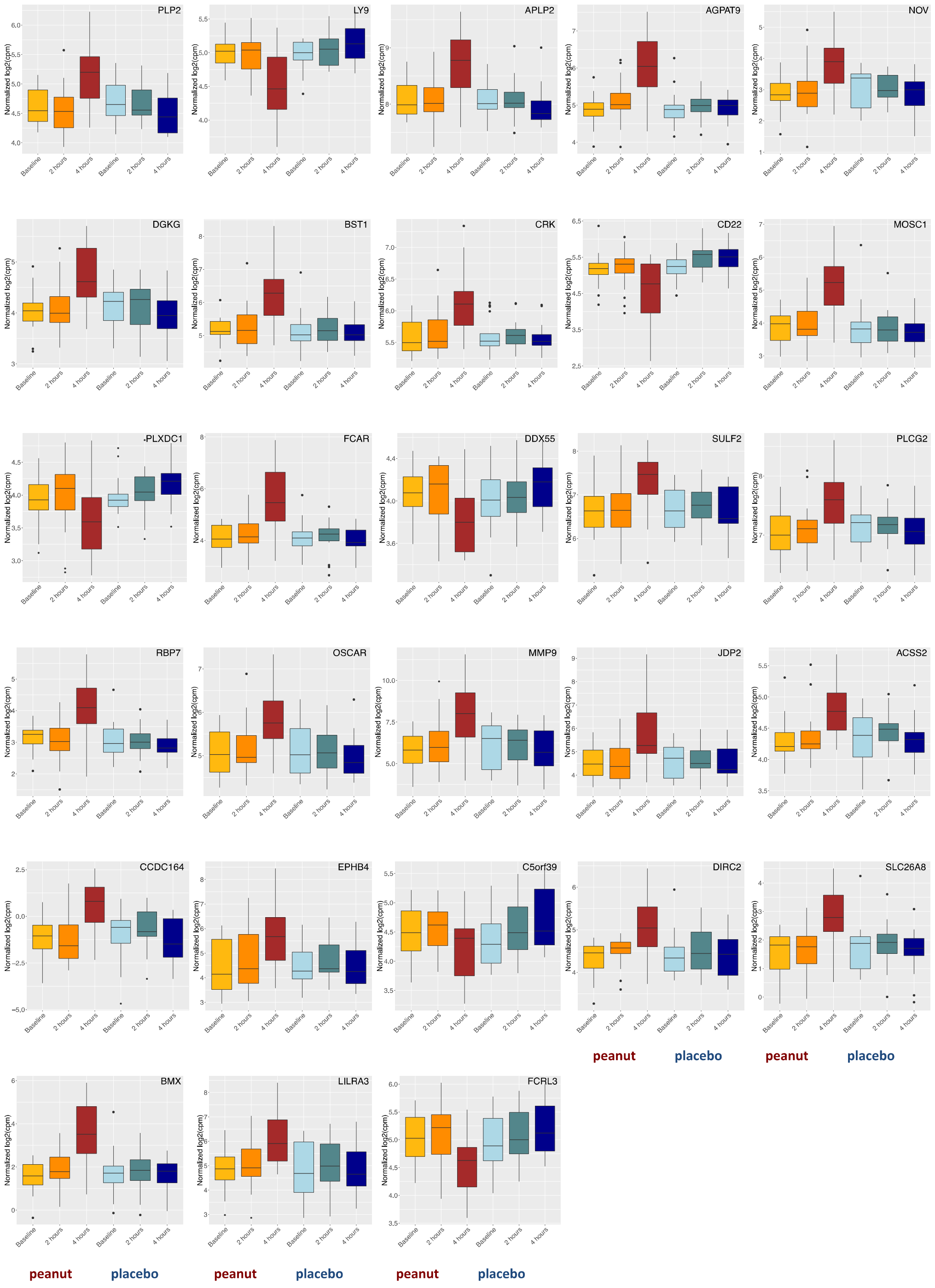

Supplementary Figure 2. **Top thirty peanut allergen response genes (peanut genes) identified by linear mixed-effects models (Bonferroni-corrected P<0.01) in the discovery cohort.** Boxplots displaying $\log_2$-cpm expression values for the top thirty peanut genes identified by linear mixed-effects model analysis (**Table 2**) excluding LTB4R and PADI4, which are included in **Fig. 2**. Gene expression levels are from the 19 peanut allergic subjects at three time points (baseline, during challenge (two hours), and end of challenge (four hours)) for both peanut and placebo challenges.

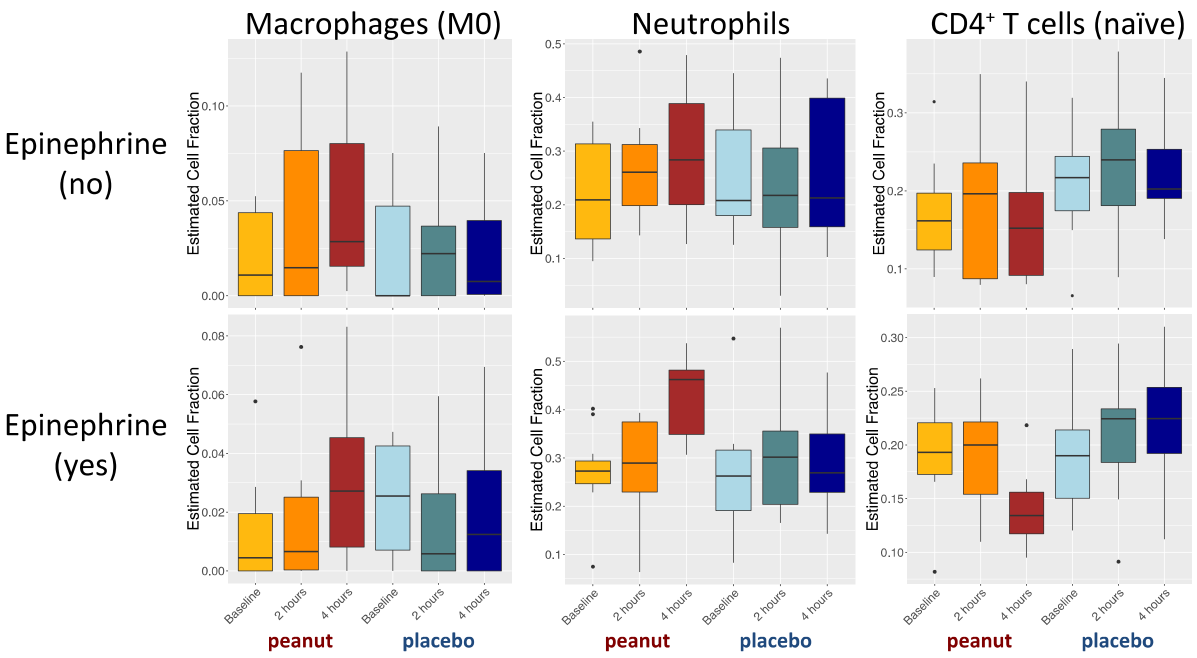

Supplementary Figure 3. **Compositional changes in leukocyte subpopulations in peanut allergic subjects, stratified by epinephrine treatment.** Boxplots displaying estimated fractions of macrophages, neutrophils, and naïve $CD4^+$ T cells at three time points (baseline, during challenge (two hours), and end of challenge (four hours)) for both peanut and placebo challenges, after stratifying the discovery cohort into subjects who received epinephrine treatment (n=11) and those who did not (n=8).

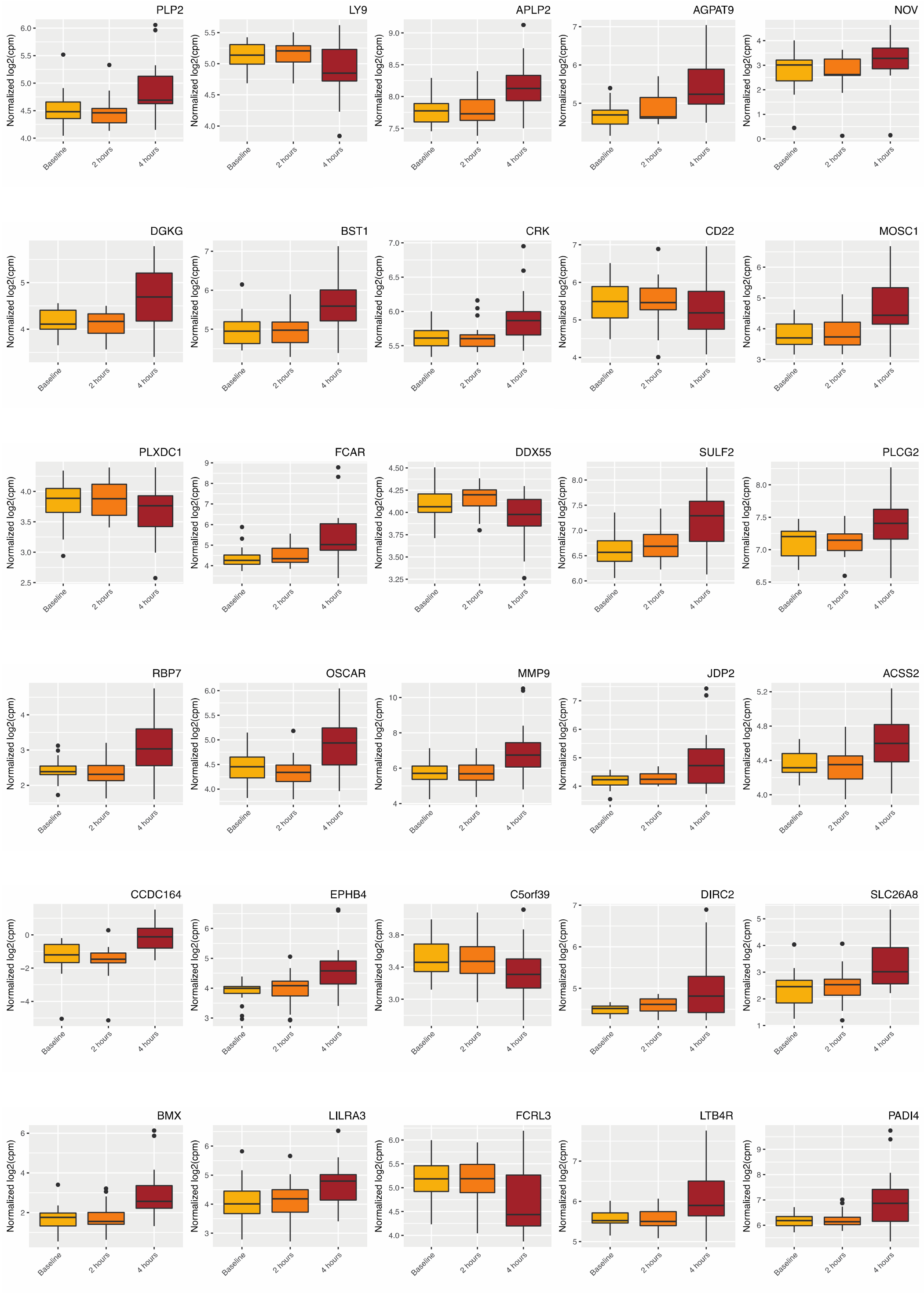

Supplementary Figure 4. **Gene expression profiles in the replication cohort of the top thirty peanut allergen response genes (peanut genes) identified in the discovery cohort.** Boxplots displaying log_2-cpm expression values for these genes in peanut allergic subjects (n=21) from the replication cohort at three time points (baseline, during peanut challenge (two hours), and end of peanut challenge (four hours)).

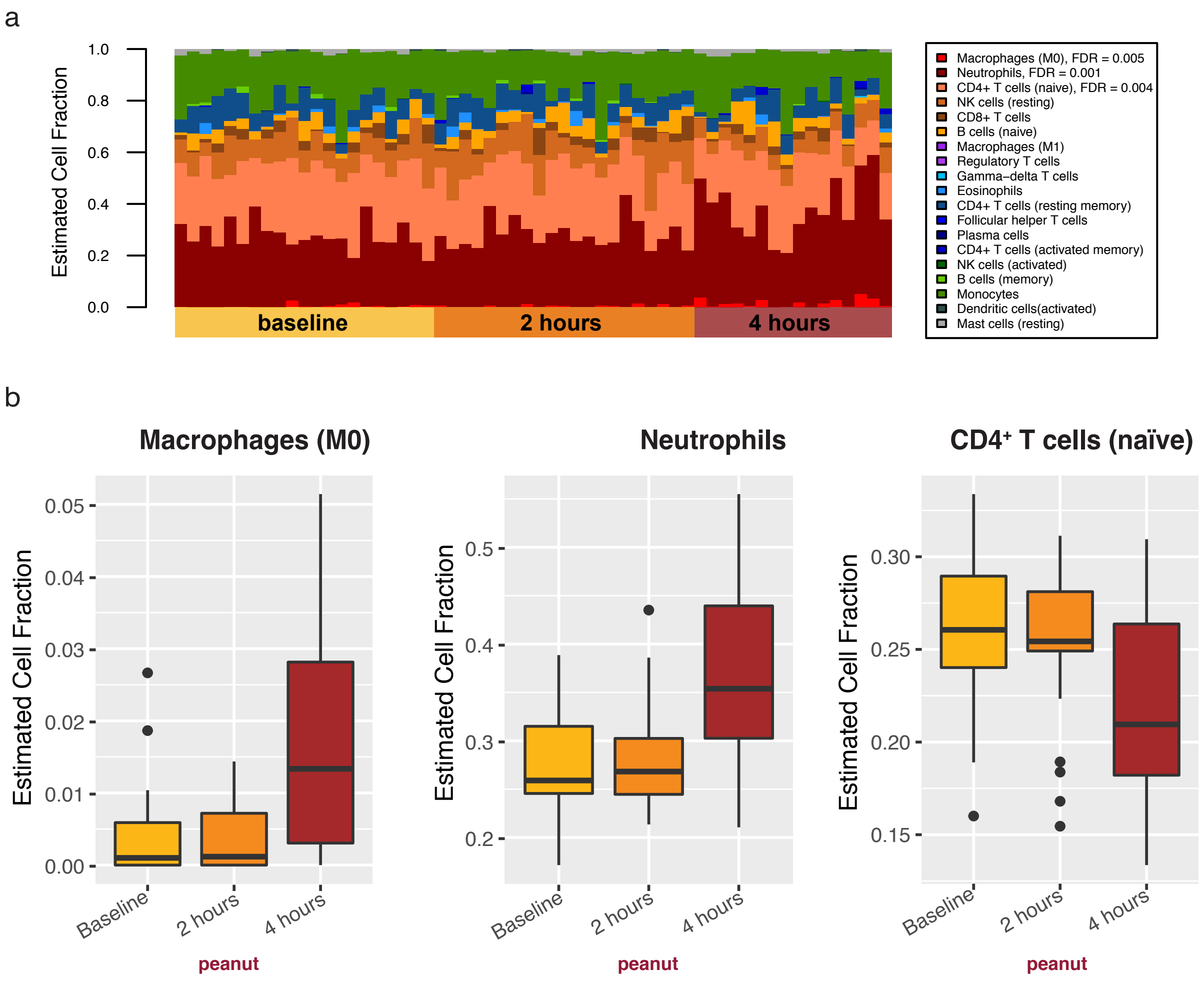

Supplementary Figure 5. **Compositional changes in leukocyte subpopulations in the replication cohort.** (A) Fractions of leukocyte subpopulations estimated from transcriptome-wide RNA-seq gene expression signatures from the replication cohort (n=21), partitioned by time point. The order in which individuals are plotted is consistent across time/challenge groups. Changes in cell type composition associated with peanut challenge were assessed using lme models. Cell types included in the analysis are indicated in the legend, listed in ranked order according to significance. FDR values are provided for the three cell types that exhibited significant changes in response to peanut but not placebo (macrophages (M0); neutrophils; naïve $CD4^{+}$ T cells). Data for these significant peanut response cell types are plotted in (B), again partitioned by time point.

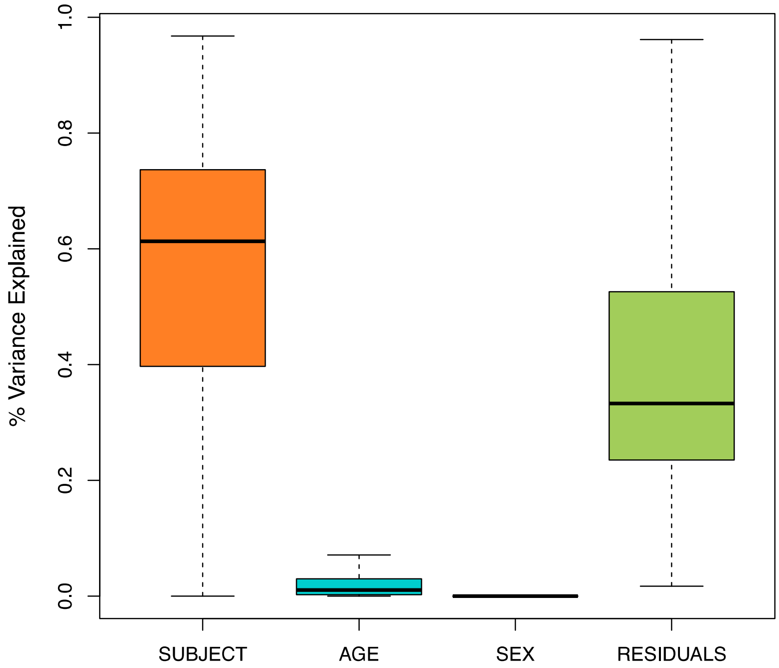

Supplementary Figure 6. Variance in gene expression explained by subject, age, and sex in the discovery cohort. Boxplots depicting the contribution of three variables on transcriptome-wide gene expression variation observed in the discovery cohort. The percent variance explained by age and sex is relatively minimal compared to that shown for subject (i.e., individual). For context, residual variation is also plotted.

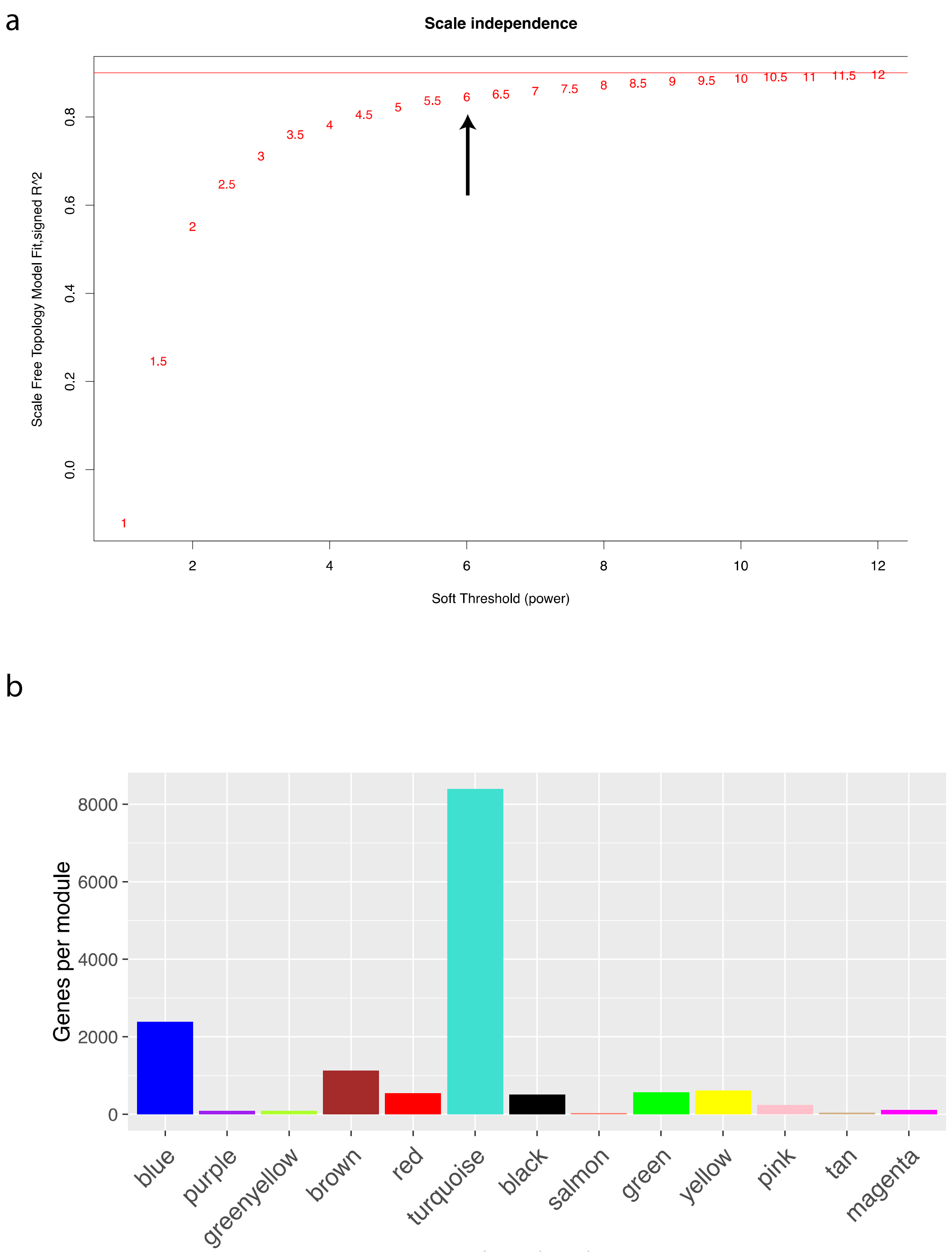

Supplementary Figure 7. **Weighted coexpression network characteristics.** (A) Scale-free topology model fit in relation to soft threshold pick, supporting beta power selection. The beta selected is indicated by the black arrow. (B) Chart depicting the number of genes in each coexpression module identified by WGCNA; each module is labeled by the color indicated on the x axis.

Supplementary Table 1. Basic functional annotations and references for the top 30 peanut genes identified by linear mixed-effects model analysis.

Gene	Functional annotation	References*
PLP2	Interacts with chemokine receptor CCR1; Involved in T-cell acute lymphoblastic leukemia	1; 2
LY9	Immunomodulatory receptor (Thymocytes/Lymphocytes); regulates T-cell differentiation;	3; 4
APLP2	Regulates expression of MHC class I	5
AGPAT9	Triglycerol synthesis; adipogenesis	6
NOV	Regulates inflammatory response, chemotaxis, and NF-KappaB	7
DGKG	Genetically associated with asthma; Macrophage differentiation	8; 9
BST1	B cell development; neutrophil adhesion and migration	10; 11
CRK	Immune regulation; NK cell-mediated cytotoxicity	12
CD22	Co-receptor expressed on B cells	13
MOSC1	Mitochondrial membrane/ enzyme	14; 15
PLXDC1	Transmembrane receptor for PEDF (anti angiogenic, anti-tumorigenic and neutrophilic function); cancer	16; 17
PADI4	Autoimmunity; inflammation	18; 19; 20
FCAR	IgA receptor; Immune signaling	21
DDX55	RNA Helicase family	22
PLCG2	Immune cell signaling/differentiation; inflammation; autoimmunity; immunodeficiency	23; 24
SULF2	Inflammation; cancer	25; 26
LTB4R	Immune cell signaling/recruitment; inflammation	27; 28
RBP7	Cellular retinol binding protein (CRBP) member	29
OSCAR	Immune cell (monocyte) signaling development; inflammation	30; 31
MMP9	Immune cell signaling/activation; inflammation	32; 33
JDP2	Cellular senescence and aging; Histone acetylation	34; 35
ACSS2	Energy generation / lipid synthesis; acetate utilization; cancer survival	36; 37; 38; 39
CCDC164	Cilia function	40
EPHB4	Thymocyte development; T cell function	41; 42
C5orf39	Apoptosis	43
SLC26A8	Anion transport; sperm motility	44; 45
DIRC2	Electrogenic lysosomal metabolite transporter	46
BMX	Immune cell signaling/differentiation; inflammation	47; 48
LILRA3	Immune cell receptor; antigen processing	49
FCRL3	Autoimmunity	50

*See Supplementary References

Supplementary Table 2. Linear mixed-effects model results from the leukocyte deconvolution analysis in the discovery cohort.

Cell Type	Chi square (LRT, Time by Trial Effect)	P value (LRT, Time by Trial Effect)	FDR (LRT, Time by Trial Effect)
Macrophages.M0	13.2398	0.0013	0.0166
Neutrophils	12.7018	0.0017	0.0166
T.cells.CD4.naive	11.6912	0.0029	0.0183
NK.cells.resting	7.0826	0.0290	0.1376
T.cells.CD8	5.0077	0.0818	0.3107
B.cells.naive	3.7562	0.1529	0.4841
Macrophages.M1	2.7354	0.2547	0.6534
T.cells.regulatory.Tregs.	2.5811	0.2751	0.6534
T.cells.gamma.delta	2.3382	0.3107	0.6558
Eosinophils	1.7906	0.4085	0.7092
T.cells.CD4.memory.resting	1.6243	0.4439	0.7092
T.cells.follicular.helper	1.6063	0.4479	0.7092
Plasma.cells	1.0831	0.5818	0.8504
T.cells.CD4.memory.activated	0.4851	0.7846	0.9950
NK.cells.activated	0.2459	0.8843	0.9950
B.cells.memory	0.1894	0.9096	0.9950
Monocytes	0.1232	0.9403	0.9950
Dendritic.cells.activated	0.0968	0.9527	0.9950
Mast.cells.resting	0.0100	0.9950	0.9950

Supplementary Table 3. Enrichment of each coexpression module for peanut genes.

Module	Total Genes in Module	Peanut Response Genes ($P<0.005$) in Module	*P value* (Fisher's exact test)	Fold-Enrichment of peanut response Genes in Module
Blue	2381	1223	1.16E-304	4.105
Purple	96	18	0.05	1.499
Greenyellow	84	11	0.485	1.047
Salmon	30	2	0.904	0.533
Tan	43	1	0.997	0.186
Red	546	43	1	0.629
Black	504	36	1	0.571
Brown	1133	92	1	0.649
Magenta	111	1	1	0.072
Pink	244	7	1	0.229
Green	570	22	1	0.308
Turquoise	8388	648	1	0.617
Yellow	610	18	1	0.236

Supplementary Table 4. Results from key driver analysis of the peanut response module. The network contained a total of 7713 genes, of which 1477 overlapped with genes in the peanut response module (N= 2381). We report here the key drivers which had an FDR ≤ 0.05.

Key Driver (KD) Gene	KD in the Gene Set? (1: Yes, 0: No)	# Target Genes	# Down-stream Genes	Layer where Gene is KD	Fold Change	*P value*	FDR
ECHDC3	1	91	146	6	3.25	$5.79x10^{-31}$	$4.47x10^{-27}$
IL1R2	1	64	90	6	3.71	$5.81x10^{-27}$	$4.48x10^{-23}$
CEBPD	0	35	47	6	3.89	$2.51x10^{-16}$	$1.94x10^{-12}$
PTENP1	0	61	116	6	2.75	$4.80x10^{-16}$	$3.70x10^{-12}$
LTB4R	1	33	52	6	3.31	$2.55x10^{-12}$	$1.97x10^{-08}$
PPP1R3D	1	19	28	6	3.54	$2.43x10^{-08}$	$1.87x10^{-04}$
PADI4	1	18	26	6	3.62	$3.54x10^{-08}$	$2.73x10^{-04}$
KLHL2	1	46	114	6	2.11	$1.11x10^{-07}$	$8.53x10^{-04}$
ATP11A	0	23	44	6	2.73	$8.24x10^{-07}$	$6.35x10^{-03}$
PHC2	1	33	44	5	3.92	$1.28x10^{-15}$	$9.87x10^{-12}$
FLOT2	1	26	45	5	3.02	$9.85x10^{-09}$	$7.60x10^{-05}$
IL18R1	1	25	43	5	3.04	$1.60x10^{-08}$	$1.23x10^{-04}$
HAL	1	38	90	5	2.20	$3.56x10^{-07}$	$2.74x10^{-03}$
HDGFRP3	0	18	29	5	3.24	$4.35x10^{-07}$	$3.35x10^{-03}$
PFKFB4	1	16	24	5	3.48	$4.68x10^{-07}$	$3.61x10^{-03}$
MAR1	0	14	21	5	3.48	$2.52x10^{-06}$	$1.95x10^{-02}$
MAPK14	1	30	43	4	3.64	$6.15x10^{-13}$	$4.74x10^{-09}$
TMCC3	1	14	16	4	4.57	$6.89x10^{-09}$	$5.31x10^{-05}$
EXOC6	0	19	30	4	3.31	$1.29x10^{-07}$	$9.98x10^{-04}$
LOC100507006	0	12	15	4	4.18	$5.98x10^{-07}$	$4.61x10^{-03}$
RAB36	0	16	27	4	3.09	$4.62x10^{-06}$	$3.56x10^{-02}$
CAMKK2	1	24	31	3	4.04	$3.29x10^{-12}$	$2.54x10^{-08}$
MAP2K6	0	15	19	3	4.12	$2.85x10^{-08}$	$2.20x10^{-04}$
GNB2	1	16	27	3	3.09	$4.62x10^{-06}$	$3.56x10^{-02}$
BMX	1	14	16	2	4.57	$6.89x10^{-09}$	$5.31x10^{-05}$
SLC9A8	1	12	17	2	3.69	$5.54x10^{-06}$	$4.27x10^{-02}$

Supplementary Table 5. Characteristics of additional food allergic subjects whose samples were included in the weighted gene coexpression network construction

**Subject**	**Suspected allergen/ food challenged**	**Sex**	**Age (years)**	**Food allergen sIgE (kU_a/L)**
FA1	Wheat	M	13	78.5
FA2	Wheat	F	10	>100
FA3	Wheat	F	9	14.3
FA4	Soy	M	6	33
FA5	Wheat	M	5	17.3
FA6	Baked Milk	M	7	18.6
FA7	Sesame	M	11	19.6

Supplementary References

1. Lee SM, et al. PLP2/A4 interacts with CCR1 and stimulates migration of CCR1-expressing HOS cells. Biochem Biophys Res Commun 324, 768-772 (2004).

2. Zhu H, Miao MH, Ji XQ, Xue J, Shao XJ. miR-664 negatively regulates PLP2 and promotes cell proliferation and invasion in T-cell acute lymphoblastic leukaemia. Biochem Biophys Res Commun 459, 340-345 (2015).

3. de la Fuente MA, et al. Molecular characterization and expression of a novel human leukocyte cell-surface marker homologous to mouse Ly-9. Blood 97, 3513-3520 (2001).

4. Chatterjee M, et al. Increased expression of SLAM receptors SLAMF3 and SLAMF6 in systemic lupus erythematosus T lymphocytes promotes Th17 differentiation. J Immunol 188, 1206-1212 (2012).

5. Peters HL, et al. Regulation of major histocompatibility complex class I molecule expression on cancer cells by amyloid precursor-like protein 2. Immunol Res 51, 39-44 (2011).

6. Shan D, et al. GPAT3 and GPAT4 are regulated by insulin-stimulated phosphorylation and play distinct roles in adipogenesis. J Lipid Res 51, 1971-1981 (2010).

7. Lin Z, et al. A novel role of CCN3 in regulating endothelial inflammation. J Cell Commun Signal 4, 141-153 (2010).

8. Melen E, et al. Analyses of shared genetic factors between asthma and obesity in children. J Allergy Clin Immunol 126, 631-637 e631-638 (2010).

9. Yamada K, Sakane F, Imai S, Tsushima S, Murakami T, Kanoh H. Regulatory role of diacylglycerol kinase gamma in macrophage differentiation of leukemia cells. Biochem Biophys Res Commun 305, 101-107 (2003).

10. Kaisho T, et al. BST-1, a surface molecule of bone marrow stromal cell lines that facilitates pre-B-cell growth. Proc Natl Acad Sci U S A 91, 5325-5329 (1994).

11. Funaro A, et al. CD157 is an important mediator of neutrophil adhesion and migration. Blood 104, 4269-4278 (2004).

12. Liu D. The adaptor protein Crk in immune response. Immunol Cell Biol 92, 80-89 (2014).

13. Kawasaki N, Rademacher C, Paulson JC. CD22 regulates adaptive and innate immune responses of B cells. J Innate Immun 3, 411-419 (2011).

14. Sparacino-Watkins CE, et al. Nitrite reductase and nitric-oxide synthase activity of the mitochondrial molybdopterin enzymes mARC1 and mARC2. J Biol Chem 289, 10345-10358 (2014).

15. Klein JM, Busch JD, Potting C, Baker MJ, Langer T, Schwarz G. The mitochondrial amidoxime-reducing component (mARC1) is a novel signal-anchored protein of the outer mitochondrial membrane. J Biol Chem 287, 42795-42803 (2012).

16. Cheng G, et al. Identification of PLXDC1 and PLXDC2 as the transmembrane receptors for the multifunctional factor PEDF. Elife 3, e05401 (2014).

17. Bagley RG, et al. Tumor endothelial marker 7 (TEM-7): a novel target for antiangiogenic therapy. Microvasc Res 82, 253-262 (2011).

18. Suzuki A, et al. Functional haplotypes of PADI4, encoding citrullinating enzyme peptidylarginine deiminase 4, are associated with rheumatoid arthritis. Nat Genet 34, 395-402 (2003).

19. Snir O, et al. Non-HLA genes PTPN22, CDK6 and PADI4 are associated with specific autoantibodies in HLA-defined subgroups of rheumatoid arthritis. Arthritis Res Ther 16, 414 (2014).

20. Spengler J, et al. Release of Active Peptidyl Arginine Deiminases by Neutrophils Can Explain Production of Extracellular Citrullinated Autoantigens in Rheumatoid Arthritis Synovial Fluid. Arthritis Rheumatol 67, 3135-3145 (2015).

21. Morton HC, Brandtzaeg P. CD89: the human myeloid IgA Fc receptor. Arch Immunol Ther Exp (Warsz) 49, 217-229 (2001).

22. Schmid SR, Linder P. D-E-A-D protein family of putative RNA helicases. Mol Microbiol 6, 283-291 (1992).

23. Ombrello MJ, et al. Cold urticaria, immunodeficiency, and autoimmunity related to PLCG2 deletions. N Engl J Med 366, 330-338 (2012).

24. Wang J, Sohn H, Sun G, Milner JD, Pierce SK. The autoinhibitory C-terminal SH2 domain of phospholipase C-gamma2 stabilizes B cell receptor signalosome assembly. Sci Signal 7, ra89 (2014).

25. Yue X. Epithelial Deletion of Sulf2 Exacerbates Bleomycin-induced Lung Injury, Inflammation and Mortality. Am J Respir Cell Mol Biol, (2017).

26. Rosen SD, Lemjabbar-Alaoui H. Sulf-2: an extracellular modulator of cell signaling and a cancer target candidate. Expert Opin Ther Targets 14, 935-949 (2010).

27. Oyoshi MK, et al. Leukotriene B4-driven neutrophil recruitment to the skin is essential for allergic skin inflammation. Immunity 37, 747-758 (2012).

28. Serezani CH, Lewis C, Jancar S, Peters-Golden M. Leukotriene B4 amplifies NF-kappaB activation in mouse macrophages by reducing SOCS1 inhibition of MyD88 expression. J Clin Invest 121, 671-682 (2011).

29. Folli C, Calderone V, Ramazzina I, Zanotti G, Berni R. Ligand binding and structural analysis of a human putative cellular retinol-binding protein. J Biol Chem 277, 41970-41977 (2002).

30. Schultz HS, et al. OSCAR-collagen signaling in monocytes plays a proinflammatory role and may contribute to the pathogenesis of rheumatoid arthritis. Eur J Immunol 46, 952-963 (2016).

31. Barrow AD, et al. OSCAR is a receptor for surfactant protein D that activates TNF-alpha release from human CCR2+ inflammatory monocytes. J Immunol 194, 3317-3326 (2015).

32. Xu L, Cai Z, Yang F, Chen M. Activation induced upregulation of MMP9 in mast cells is a positive feedback mediator for mast cell activation. Mol Med Rep 15, 1759-1764 (2017).

33. Parks WC, Wilson CL, Lopez-Boado YS. Matrix metalloproteinases as modulators of inflammation and innate immunity. Nat Rev Immunol 4, 617-629 (2004).

34. Huang YC, Saito S, Yokoyama KK. Histone chaperone Jun dimerization protein 2 (JDP2): role in cellular senescence and aging. Kaohsiung J Med Sci 26, 515-531 (2010).

35. Murata T, et al. Involvement of Jun dimerization protein 2 (JDP2) in the maintenance of Epstein-Barr virus latency. J Biol Chem 286, 22007-22016 (2011).

36. Hur H, Kim YB, Ham IH, Lee D. Loss of ACSS2 expression predicts poor prognosis in patients with gastric cancer. J Surg Oncol 112, 585-591 (2015).

37. Lakhter AJ, Hamilton J, Konger RL, Brustovetsky N, Broxmeyer HE, Naidu SR. Glucose-independent Acetate Metabolism Promotes Melanoma Cell Survival and Tumor Growth. J Biol Chem 291, 21869-21879 (2016).

38. Schug ZT, et al. Acetyl-CoA synthetase 2 promotes acetate utilization and maintains cancer cell growth under metabolic stress. Cancer Cell 27, 57-71 (2015).

39. Comerford SA, et al. Acetate dependence of tumors. Cell 159, 1591-1602 (2014).

40. Wirschell M, et al. The nexin-dynein regulatory complex subunit DRC1 is essential for motile cilia function in algae and humans. Nat Genet 45, 262-268 (2013).

41. Jin W, Luo H, Wu J. Effect of reduced EPHB4 expression in thymic epithelial cells on thymocyte development and peripheral T cell function. Mol Immunol 58, 1-9 (2014).

42. Lu P, Shih C, Qi H. Ephrin B1-mediated repulsion and signaling control germinal center T cell territoriality and function. Science 356, (2017).

43. Xiong Y, et al. Annexin II receptor induces apoptosis independent of Annexin II. Apoptosis 18, 925-939 (2013).

44. Lohi H, et al. Functional characterization of three novel tissue-specific anion exchangers SLC26A7, -A8, and -A9. J Biol Chem 277, 14246-14254 (2002).

45. Dirami T, et al. Missense mutations in SLC26A8, encoding a sperm-specific activator of CFTR, are associated with human asthenozoospermia. Am J Hum Genet 92, 760-766 (2013).

46. Savalas LR, et al. Disrupted in renal carcinoma 2 (DIRC2), a novel transporter of the lysosomal membrane, is proteolytically processed by cathepsin L. Biochem J 439, 113-128 (2011).

47. Boucheron N, Ellmeier W. The role of Tec family kinases in the regulation of T-helper-cell differentiation. Int Rev Immunol 31, 133-154 (2012).

48. Cenni B, Gutmann S, Gottar-Guillier M. BMX and its role in inflammation, cardiovascular disease, and cancer. Int Rev Immunol 31, 166-173 (2012).

49. Cella M, et al. A novel inhibitory receptor (ILT3) expressed on monocytes, macrophages, and dendritic cells involved in antigen processing. J Exp Med 185, 1743-1751 (1997).

50. Tsubata T. Role of inhibitory BCR co-receptors in immunity. Infect Disord Drug Targets 12, 181-190 (2012).
